# Spatial de-concentration of fatal and nonfatal firearm violence in Boston, MA, 2007–2021

**DOI:** 10.1186/s40621-025-00572-2

**Published:** 2025-03-24

**Authors:** Faizah Shareef, Emma L. Gause, Suzanne McLone, Erika Gebo, Jonathan Jay

**Affiliations:** 1 University of California, San Diego, CA USA; 2 Center for Climate and Health, Boston University School of Public Health, Boston, MA USA; 3 Firearm Injury & Policy Research Program, Department of Pediatrics, School of Medicine, University of Washington, Seattle, WA USA; 4 Department of Epidemiology, Boston University School of Public Health, Boston, MA USA; 5 Department of Sociology & Criminal Justice, Suffolk University, Boston, MA USA; 6 Department of Community Health Sciences, Boston University School of Public Health, 801 Massachusetts Avenue Crosstown Center Rm 444, Boston, MA 02118 USA

## Abstract

**Background:**

It is a “law” of criminology that urban crime chronically recurs at the same microplaces (i.e., street segments and intersections). An influential study found high concentrations of firearm violence at microplaces in Boston, MA, from 1980 to 2008. The current study assessed whether this strong spatial concentration has persisted.

**Approach:**

Fatal and nonfatal shooting incidents with one or more victims from January 2007 through September 2021 were included, obtained from the Boston Police Department. We matched shootings to the closest microplaces, i.e., intersections and street segments in Boston (*n* = 32,267). We operationalized spatial concentration as the probability of shootings occurring at the same microplace. We employed a case-only design, with shootings as the units of analysis; the outcome of interest was a binary indicator for whether another shooting in the dataset occurred at the same microplace in the past or future. We used log-linear regression to estimate this outcome as a function of study year.

**Results:**

Annual shootings decreased over the study period, except for a spike in 2020. Spatial concentration of shootings declined from 62% in 2007 to 55% in 2021. We estimated that spatial concentration declined by an average of 1.8% per year [95% CI (-1.1, -3.4), *p* < 0.001].

**Implications:**

This declining trend in the spatial concentration of firearm violence has important implications for place-based interventions and underscores the need to monitor this trend over time. Social media, which reshapes social interactions and is linked to community violence, warrants further study as a potential cause.

**Supplementary Information:**

The online version contains supplementary material available at 10.1186/s40621-025-00572-2.

## Introduction

Through the late 1980s and early 1990s there was a spike in firearm violence across United States cities, followed by a decline and leveling off from the mid-1990s through 2010 [1–3]. During this period, there was a growing awareness that crime data were clustered at a small subset of microplaces, i.e., small spatial units, such as street segments and intersection [4], and that the persistence or volatility of these “hot spots” explained a large proportion of citywide trends in crime data over time [5]. These studies prompted scholars to posit the “law of crime concentration at place [6].”

Spatial concentration also emerged as an important characteristic of firearm violence. An influential study by Braga and colleagues found that declining incidence at a small subset of microplaces accounted for firearm violence reductions in Boston, MA from 1980 to 2008 [7]. In that analysis, the 5% of microplaces where crime rates fluctuated most dramatically over time accounted for 74% of the firearm violence decline from 1980 to 2008. Researchers subsequently found similar concentration of firearm violence at microplaces in other U.S. cities, e.g., Indianapolis, IN [8] and Minneapolis, MN [9].

This microplace-focused thinking has guided place-based prevention strategies such as surveillance cameras [10] and “hot spots policing,” [11, 12] along with best practice guidance [13] and federal grant proposal recommendations. Some but not all, research has supported the efficacy of these interventions for reducing violence, though estimated effect sizes have been modest [14]. However, this spatially targeted approach to violence prevention has evident downsides: most areas characterized as hot spots are racially, ethnically, and economically marginalized, leading to more surveillance and police encounters in areas that are already targeted and overpoliced [14, 15].

Given these tradeoffs, it is essential to reexamine whether high levels of spatial concentration in urban firearm violence persist. Societal changes since the early 2000s (e.g., gentrification [16], social media [17, 18], and other factors) could have influenced the spatial distribution of violence; yet little recent work has examined temporal trends in the spatial concentration of firearm violence. The current study revisits these trends in Boston, MA, where earlier research contributed to the dominant understanding of spatial concentration [7]. Assessing the persistence of spatial concentration is necessary to balance the benefits and harms of place-based practices and to determine whether strategies and guidelines should be revised.

## Methods

Firearm incident data were provided by the Boston Police Department (BPD) for the period between January 2007 through September 2021. Studying this time period enabled us to assess changes from a baseline that included the end of the study period from Braga and colleagues’ original work (i.e., 1990–2008), while capturing any possible changes during the first year and a half of the COVID-19 pandemic. All incidents occurred within the city of Boston. The study outcomes were fatal and nonfatal shooting incidents with one or more victims (*n* = 3,342). One incident was excluded for unknown location, yielding a final analytic sample of *n* = 3,341 shootings.

Data were cleaned and geocoded by the BPD. We assigned shootings to geographical intersections when the location was based on cross streets (e.g., “South St. & Main St.”); otherwise, we assigned shootings to the street segment corresponding to the address provided. For this investigation, we considered all microplaces (*n* = 31,152) across Boston, defined as all street segments (*n* = 19,071) and intersections (*n* = 12,081), mirroring the design of Braga et al. [12] (Table 1).

**Table 1:** Summary statistics

	*N*	%
Microplaces	31,152	–
Street segments	19,071	61.2
Intersections	12,081	38.8
Shootings (1/1/2007 through 9/21/2021)	3,341	–
Shootings at repeat locations	1,882	56.3
Shootings at non-repeat locations	1,459	43.7

We operationalized spatial concentration as the probability that for any given shooting, another shooting at the same microplace would appear in our dataset over a 14.75-year period. Every shooting in the dataset was assigned a binary indicator for whether it occurred at the same location as any other shootings in the dataset. Thus, this *repeat* indicator was set to 0 for all shootings that occurred at unique locations and to 1 for shootings where the shooting’s location appeared at least twice in the data. This binary approach was tailored to the fundamental question of whether prevention resources would be well-directed to locations based on their recent history of firearm violence incidence.

We analyzed whether the probability of the *repeat* outcome changed over time within the case-series. This case-only approach provides a valid strategy for assessing etiological heterogeneity among cases, especially when non-cases lack the relevant characteristics to serve as controls (i.e., only shootings can be repeats or non-repeats for our outcome of interest) [19]. Using log-linear regression, we modeled the probability of *repeat* = 1 as a linear function of study year. In other words, we estimated the year-over-year time trend in spatial concentration.

To test whether the study design or exogenous time trends might have introduced spurious patterns in the *repeat* indicator, we conducted Monte Carlo simulations in which we randomly reassigned the location of each shooting while preserving the shooting’s timing. For each of these trials, we recalculated the *repeat* indicator and re-estimated the parameters of the logistic regression model using the simulated dataset. We compared the estimated time trend from our real data to the time trends within the simulated data over 10,000 trials.

## Results

**Fig. 1:**
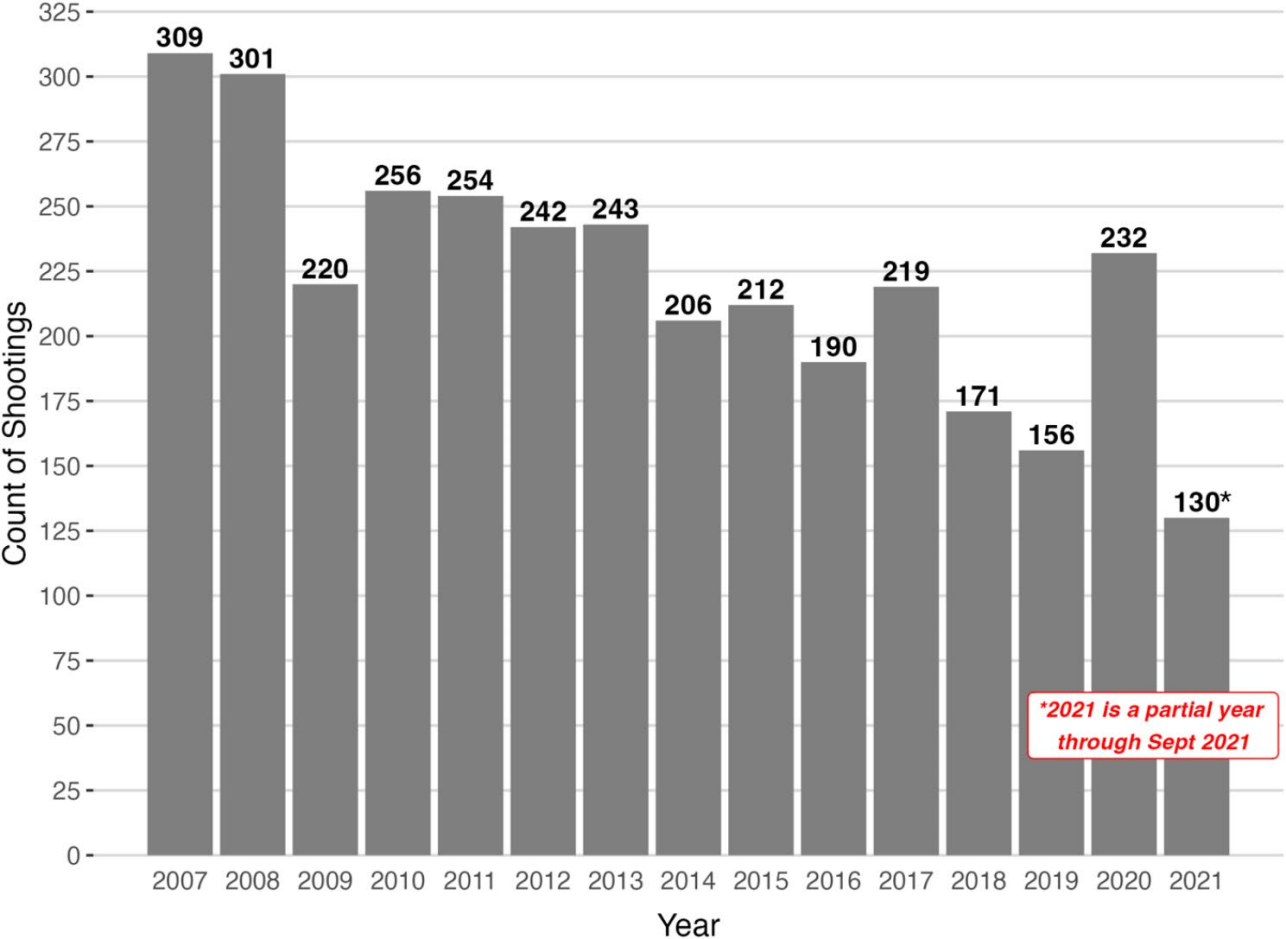
Annual shooting counts in Boston, MA: January 2007-September 2021

**Fig. 2:**
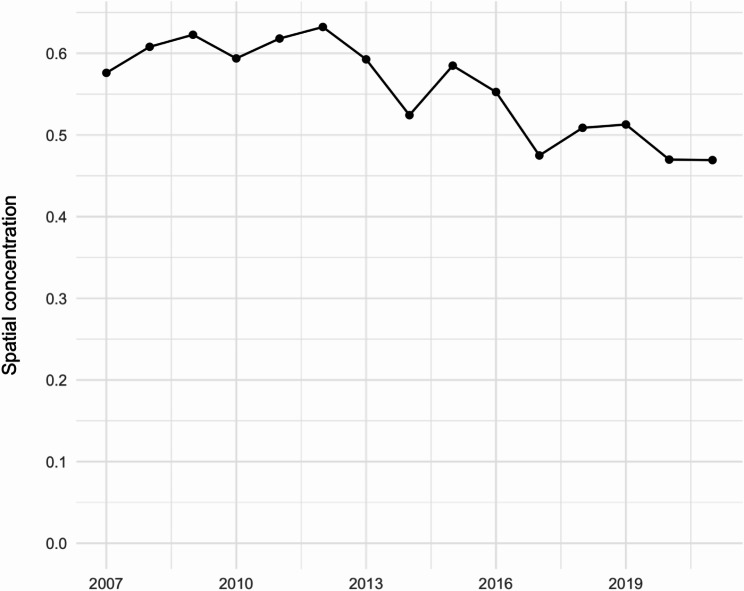
Spatial concentration of firearm violence at microplaces in Boston, MA: January 2007-September 2021. Note: Spatial concentration is defined as the probability that for any given shooting, another shooting at the same microplace would appear in our dataset

**Fig. 3:**
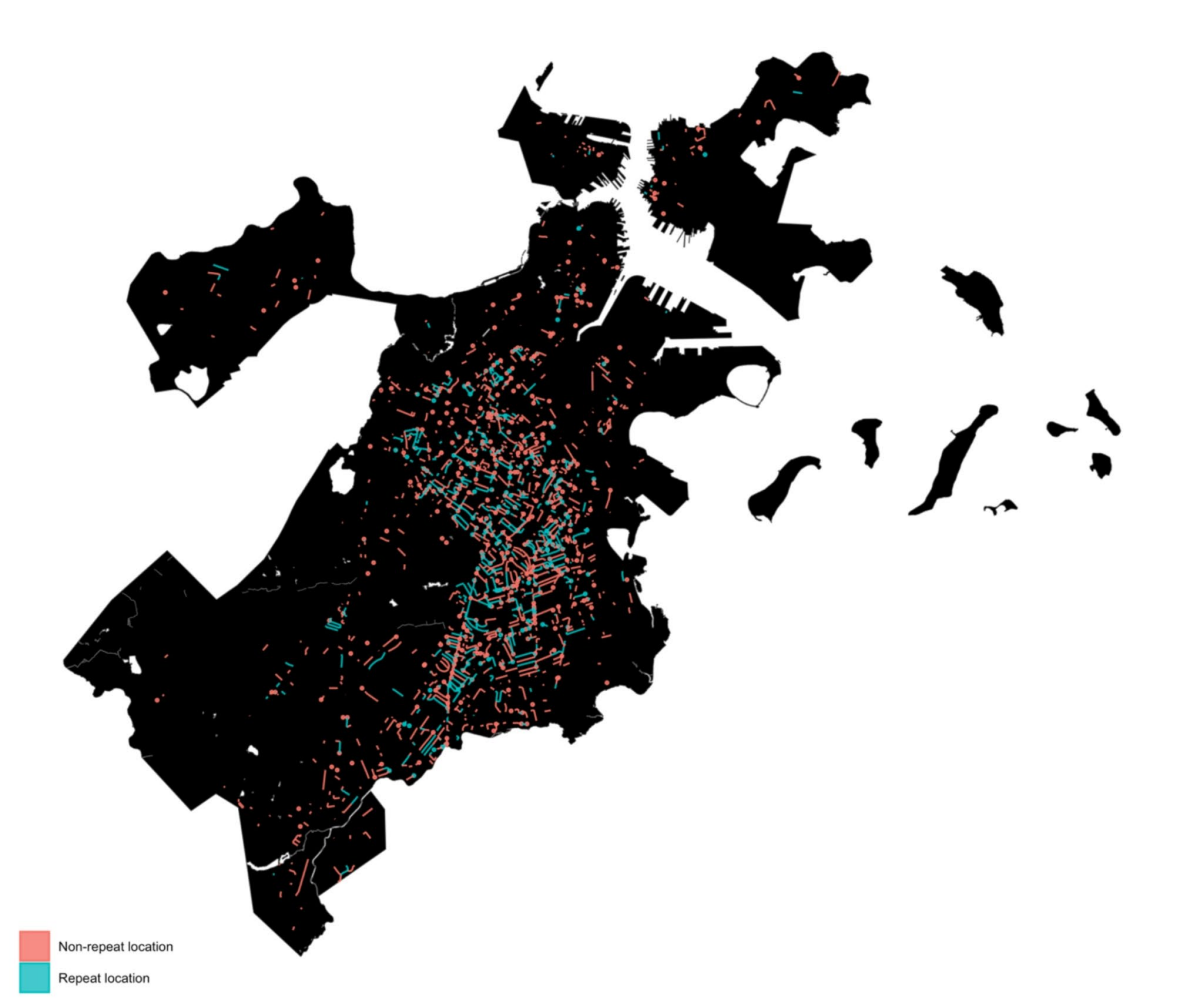
Spatial distribution of repeat and non-repeat shooting locations in Boston, MA, 2007–2021

Overall, annual shootings decreased during the study period from *n* = 309 in 2007 to 156 in 2019, though there was a spike in shootings incidence in 2020 (Fig. 1), our last full year of data, when firearm violence grew nationally amidst the COVID pandemic and highly visible police violence against Black individuals. Spatial concentration of shootings declined from 57.6% in 2007 to 46.9% in 2021, with an interim/one-off peak at 63.2% in 2012. In other words, more than half of the shootings in 2007 occurred at locations that experienced at least one other shooting during the study period but less than half of 2021 shootings showed this characteristic. Despite changes in total firearm shootings incidence during 2020, we did not see an accompanying increase in spatial concentration. The trend of decreasing spatial concentration over time was approximately linear and monotonic (Fig. 2). Repeat locations and non-repeat locations were distributed across Boston neighborhoods, especially the crescent of neighborhoods with the highest firearm violence incidence (Fig. 3).

We estimated that spatial concentration declined on average by 1.8% per year (95% CI [−1.1, −3.4], *p* < 0.001) throughout the 14.75-year study period (Table 2). In relative terms, this model-estimated change from 2007 to 2021 aggregates to a 22.1% reduction in spatial concentration. In absolute terms, it is a 13.8% point reduction, equal to shifting 31.7 shootings from *repeat* to *non-repeat* locations in a year with average annual shootings levels. None of the 10,000 Monte Carlo simulations yielded a time trend of equal magnitude in any direction (*p* < 0.0001), suggesting that this patterning was not introduced by the study design or exogenous time trends.

**Table 2:** Regression model results

	Relative Risk	95% CI	Conventional *p*-value	Monte Carlo *p*-value
Year	0.982	0.976, 0.989	< 0.001	< 0.0001

Note: Monte Carlo p value is based on comparison of the likelihood of the regression coefficient in observed data, versus the coefficient of the same model fit to data in which date-location pairs were randomly reshuffled, for 10,000 iterations

## Discussion

This brief report finds that a modest, steady de-concentration of firearm violence occurred between 2007 and 2021 in Boston, MA, even continuing through the COVID-related spike in 2020 [20]. While substantial spatial concentration persisted at the end of the study period, to our knowledge, this is the first study to identify a significant, long-term decline in the citywide spatial concentration of firearm violence at microplaces.

This finding raises important questions for the science and practice of firearm violence prevention. Our study design does not permit us to attribute causation, but several explanations stand out as most likely due to the steady, long-term deconcentration trend we found. One possibility is that place-based prevention strategies have effectively reduced the probability of shootings recurring at the same location, and that these program effects have steadily accumulated over time. Another possibility is that the social forces of gentrification and displacement, which have pushed economically marginalized residents out of some neighborhoods and often outside city limits, have contributed to shifting spatial patterns in firearm violence [16].

A third key possibility is that the rise of social media and other digital interactions have contributed to the prolonged, nearly linear changes we observed [21]. Our study period closely coincides with the timing of linear growth in active users of YouTube and Facebook [22]. The escalation of interpersonal disputes through online interactions is believed to have a large, growing influence on community firearm violence [17]. Digital interactions could facilitate the spatial de-concentration of firearm violence by enabling individuals to share their locations, either intentionally or unintentionally, with others (e.g., “dropping a [GPS] pin” or posting a photograph from a recognizable location) [21, 23]. Given these behaviors, violence risk is less confined to the subset of locations where individuals are known to live or spend time routinely; many more locations become potential conflict sites.

If the decline in spatial concentration continues, violence prevention programs may require less attention to microplace “hotspots.” Some place-based interventions, such as vacant lot greening, reduce violence over larger geographical areas than microplaces [24], potentially because they improve neighborhood-level social processes [25]. Community-based violence intervention (CVI) programs, such as street outreach, are more focused on individuals, groups, and communities than on narrowly defined physical locations. Moreover, some CVI programs have adopted online intervention models [26].

More spatially precise place-based interventions, such as surveillance cameras and flood lighting [27], would be decreasingly aligned with the problem as firearm violence becomes less spatially concentrated. However, any harms associated with these interventions, e.g., their contributions to the over-policing of racially and ethnically minoritized populations [28, 29], would likely not decline. Thus, the spatial de-concentration of firearm violence arguably supports increased investment in community-level, public health approaches to firearm violence prevention such as greening interventions and CVI programs.

### Limitations

Our results cannot be generalized to cities other than Boston, MA, which we analyzed here. Our study period also ends in September 2021, though shooting levels in Boston resumed their long-term downward trajectory in 2022, so the observed trends likely continued past the study period.

The overall downward trajectory of firearm violence levels in Boston suggests that repeat shootings at microplaces may not have been displaced to other locations in the city. However, spatiotemporal modeling could provide important additional information about changes in location and timing of firearm violence [30].

Our descriptive analysis does not offer explanations for the phenomenon we identify in this study. Future research could test potential hypotheses, e.g., by incorporating data on place-based intervention dosage, gentrification patterns, or data on digital interactions.

## Conclusion

To our knowledge, no other study has assessed recent trends in the spatial concentration of firearm violence in a major U.S. city. We found that the spatial concentration of shootings in Boston has modestly but steadily declined since the period covered by prior work. Our study also suggests that the ‘law’ of spatial concentration of violence may be weakening over time; future studies will be needed to continue examine these shifts. One possible factor is the widespread uptake in the use of social media and its role in escalating disputes and sharing user locations. However, the factors driving this shift are still unclear and require future studies. If the importance of microplace “hotspots” is waning, community-level violence interventions may need to focus more on interpersonal dynamics (including online interactions) and place-based strategies at higher ecological levels (e.g., neighborhoods). Deepening our understanding of how these trends impact neighborhoods and communities can inform future interventions.

## Supplementary Information

Additional file1

## Data Availability

No datasets were generated or analysed during the current study.

## References

[1] Blumstein A. Youth violence, guns, and the illicit-drug industry. J Crim L Criminol. 1995;86:10.

[2] Cook PJ, Laub JH. The unprecedented epidemic in youth violence. Crime Justice. 1998;24:27–64.

[3] Cook PJ, Laub JH. After the epidemic: recent trends in youth violence in the united States. Crime Justice. 2002;29:1–37.

[4] Sherman LW, Gartin PR, Buerger ME. Hot spots of predatory crime: routine activities and the criminology of place. Criminology. 1989;27(1):27–56.

[5] Weisburd D, et al. Trajectories of crime at places: A longitudinal study of street segments in the City of Seattle. Criminology. 2004;42(2):283–322.

[6] Weisburd D. The law of crime concentration and the criminology of place. Criminology. 2015;53(2):133–57.

[7] Braga AA, Papachristos AV, Hureau DM. The concentration and stability of gun violence at micro places in Boston, 1980–2008. J Quant Criminol. 2010;26(1):33–53.

[8] Magee LA. Community-Level social processes and firearm shooting events: A multilevel analysis. J Urb Health. 2020;97(2):296–305. 10.1007/s11524-020-00424-y PMC7101464 32107724

[9] Koper C.S., Egge S.J., C., Lum. Institutionalizing Place-Based approaches: opening ‘cases’ on gun crime hot Spots1. Policing: J Policy Pract. 2015;9(3):242–54.

[10] Welsh BC, Farrington DP. Effects of closed circuit television surveillance on crime. Campbell Syst Reviews. 2008;4(1):1–73.

[11] Sherman LW, Weisburd D. General deterrent effects of Police patrol in crime hot spots: A randomized, controlled trial. Justice Q. 1995;12(4):625–48.

[12] Braga AA, Weisburd DL. Policing problem places: crime hot spots and effective prevention. Oxford University Press; 2010.

[13] Programs OoJ. *CrimeSolutions.gov*. 2024; Available from: https://crimesolutions.ojp.gov/

[14] Braga AA, Papachristos AV, Hureau DM. The effects of hot spots policing on crime: an updated systematic review and meta-analysis. Justice Q. 2014;31(4):633–63.

[15] Neil R, MacDonald JM. Where Racial and ethnic disparities in policing come from: the Spatial concentration of arrests across six cities. Criminol Public Policy. 2023;22(1):7–34. 10.1111/1745-9133.12603 PMC13384522 42518991

[16] Spitzer SA, et al. Gentrification as a factor in the incidence of firearm injuries. JAMA Surg. 2023;158(11):1152–8. 37728889 10.1001/jamasurg.2023.3939 PMC10512160

[17] Hyatt JM, Densley JA, Roman CG. Social media and the variable impact of violence reduction interventions: Re-Examining focused deterrence in Philadelphia. Social Sci. 2021;10(5):147.

[18] Patton DU, et al. Social media as a vector for youth violence: A review of the literature. Comput Hum Behav. 2014;35:548–53.

[19] Rundle AG, et al. Causal inference with Case-Only studies in injury epidemiology research. Curr Epidemiol Rep. 2022;9(4):223–32. 37152190 10.1007/s40471-022-00306-8 PMC10161782

[20] Pino EC, et al. Trends in violent penetrating injuries during the first year of the COVID-19 pandemic. JAMA Netw Open. 2022;5(2):e2145708. 35133435 10.1001/jamanetworkopen.2021.45708 PMC8826178

[21] Patton DU, et al. Sticks, stones and Facebook accounts: what violence outreach workers know about social media and urban-based gang violence in Chicago. Comput Hum Behav. 2016;65:591–600.

[22] Ritchie H. *The Rise of Social Media*. 2023 [cited 2024; Available from: https://ourworldindata.org/rise-of-social-media

[23] Stuart F. Code of the tweet: urban gang violence in the social media age. Soc Probl. 2019;67(2):191–207.

[24] Branas CC, et al. Citywide cluster randomized trial to restore blighted vacant land and its effects on violence, crime, and fear. Proc Natl Acad Sci U S A. 2018;115(12):2946–51. 29483246 10.1073/pnas.1718503115 PMC5866574

[25] Aiyer SM, et al. From broken windows to busy streets: a community empowerment perspective. Health Educ Behav. 2015;42(2):137–47. 25512073 10.1177/1090198114558590

[26] Sichel CE, et al. Leveraging youths’ digital literacies: the E-Responder social media violence interruption model and pilot evaluation. J Prev Interv Community. 2019;47(2):76–89. 30907278 10.1080/10852352.2019.1582145

[27] Chalfin A, et al. Reducing crime through environmental design: evidence from a randomized experiment of street lighting in new York City. J Quant Criminol. 2022;38(1):127–57.

[28] Kramer R, Remster B. Stop, Frisk, and assault?? Racial disparities in Police use of force during investigatory stops. Law Soc Rev. 2018;52(4):960–93.

[29] Fagan J, et al. Stops and stares: street stops, surveillance, and race in the new policing mental health, the law, & the urban environment. Fordham Urban Law J. 2016;43(3):539–614.

[30] Nsoesie EO, et al. Mapping disparities in homicide trends across Brazil: 2000–2014. Injury Epidemiol. 2020;7:1–11. 10.1186/s40621-020-00273-y PMC7487619 32892747

