# Supplementary material for: Spatial de-concentration of fatal and nonfatal firearm violence in Boston, MA, 2007–2021
Source: Inj Epidemiol. 2025 Mar 24;12:18. doi: 10.1186/s40621-025-00572-2 (PMC11931767; doi:10.1186/s40621-025-00572-2)

**Supplemental Figure 1.** Annual shooting counts in Boston, MA: January 2007-September 2021 by repeat vs. non-repeat location.


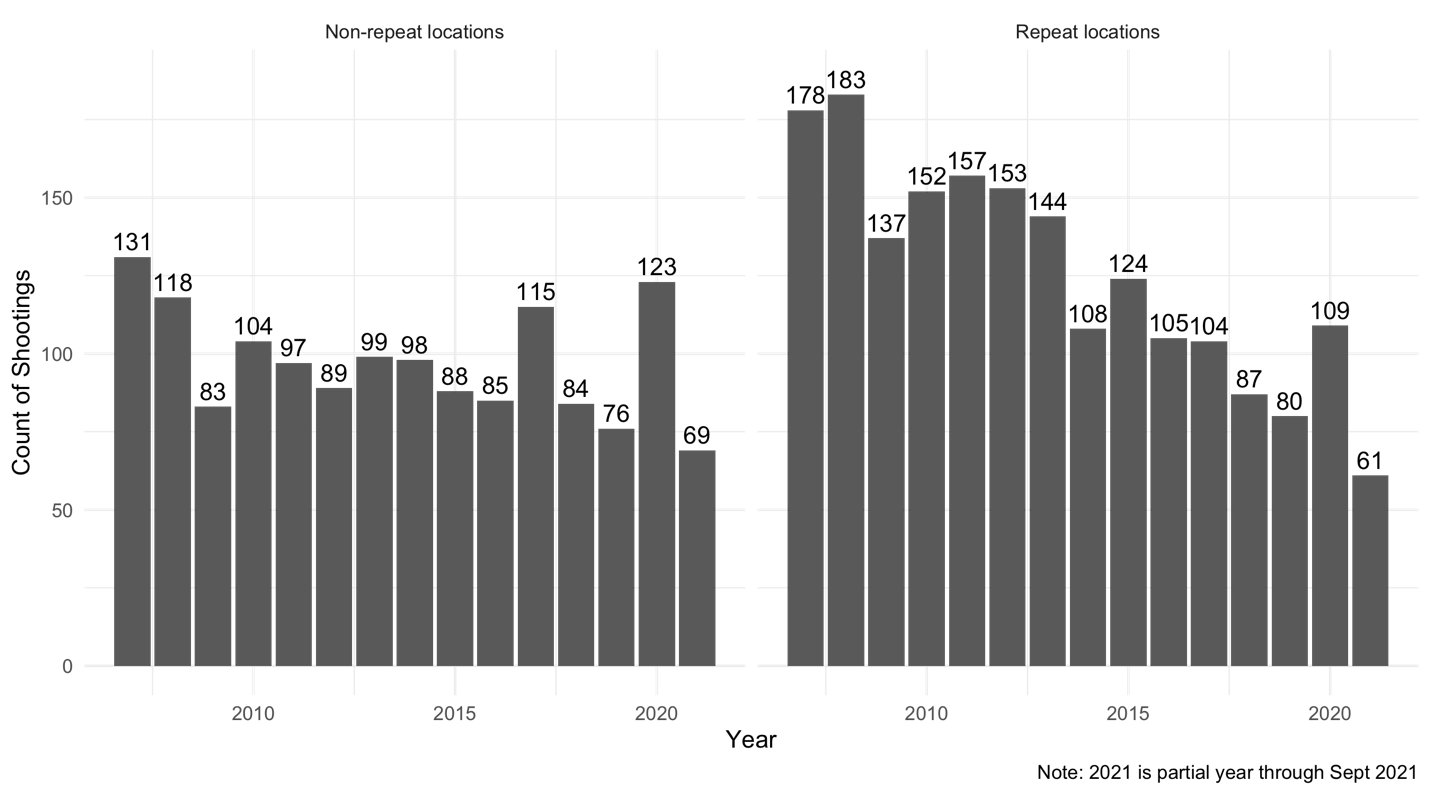

Supplement: Supplementary file 1 — Additional file1 [file 40621_2025_572_MOESM1_ESM.docx]
